# Impact of perioperative factors on nadir serum prostate‐specific antigen levels after holmium laser enucleation of prostate

**DOI:** 10.1002/bco2.68

**Published:** 2021-01-05

**Authors:** Mary Martos, Jonathan E. Katz, Madhumita Parmar, Anika Jain, Nachiketh Soodana‐Prakash, Sanoj Punnen, Mark L. Gonzalgo, Feng Miao, Isildinha M. Reis, Nicholas Smith, Hemendra N. Shah

**Affiliations:** ^1^ Miller School of Medicine University of Miami Miami FL USA; ^2^ Department of Urology Miller School of Medicine University of Miami Miami FL USA; ^3^ Department of Urology Sylvester Comprehensive Cancer Center Miller School of Medicine University of Miami Miami FL USA; ^4^ Division of Biostatistics Department of Public Health Sciences Sylvester Biostatistics and Bioinformatics Shared Resource Miller School of Medicine University of Miami Miami FL USA

**Keywords:** endoscopic enucleation for prostate, HoLEP, laser prostatectomy, prostate cancer screening, serum PSA, transurethral resection of prostate

## Abstract

**Objective:**

To investigate the relationship of preoperative prostate size, urinary retention, positive urine culture, and histopathological evidence of prostatitis or incidental prostate cancer on baseline and 3‐month nadir prostate‐specific antigen (PSA) value after Holmium laser enucleation of prostate (HoLEP).

**Patients and methods:**

Data from 90 patients who underwent a HoLEP by En‐bloc technique were analyzed. PSA values at baseline and at 3‐month follow‐up, preoperative urinary retention and urine culture status, weight of resected tissue, and histopathological evidence of prostatitis or prostate cancer were recorded. We performed univariable and multivariable gamma‐regression analyses to determine the impact of the aforementioned perioperative variables on preoperative PSA, 3‐month postoperative PSA, and change in PSA.

**Results:**

Serum PSA reduced significantly at 3 months from 6.3 ± 5.9 ng/mL to 0.6 ± 0.6 ng/mL. On both univariable and multivariable analysis, 3‐month nadir level was independent of all preoperative factors examined, except preoperative urinary retention status. Although patients with smaller prostate (resected tissue weight <40 g) had less percentile reduction in PSA when compared with those with larger prostate (resected tissue weight >80 g) (77.67% vs 89.06%; *P* < .001), patients from both these groups noted a similar PSA nadir level after 3 months (0.54 vs 0.56 ng/dL). The drop in PSA level after HoLEP remained stable up to 1‐year follow‐up.

**Conclusions:**

PSA nadir 3 months after HoLEP remains relatively consistent across patients, regardless of preoperative prostate size, PSA value, urine culture status, and histopathological evidence of prostatitis or incidental prostate cancer.

**Abbreviations**
BPH: benign prostatic hyperplasia
HoLEP: holmium laser enucleation of prostate
Pca: prostate cancer
PSA: serum PSA level
TURP: transurethral resection of prostate

## 1 INTRODUCTION

Reduction in Prostate‐Specific Antigen (PSA) following resection of prostate for benign prostatic hyperplasia (BPH) is directly proportional to the volume of adenoma removed.^1^, ^2^, ^3^, ^4^ Since all minimally invasive therapies do not remove the same volume of adenoma, PSA nadir will differ based on the procedure used to treat BPH. Since PSA plays an important role in prostate cancer (Pca) screening, it is necessary to have an adjusted normal PSA nadir for men with a history of adenomectomy for BPH.^5^

Holmium laser enucleation of the prostate (HoLEP) has become the standard endoscopic enucleation techniques for surgical treatment of BPH. Although enucleation is well documented to result in dramatic reduction in PSA levels, the nadir level ranges widely from 0.9 to 1.9 ng/dL at 3‐6 months post‐procedure.^6^, ^7^, ^8^ Elmansy HM proposed that if post‐HoLEP PSA reduction is <50%, these patients should be followed with frequent PSA measurements to allow earlier detection of Pca.^6^ Recent studies also recommend prostatic biopsy for all patients with post‐HoLEP PSA above 1 ng/dL.^9^

If one attempts to calculate an expected nadir level of PSA based on formula of percent reduction, the nadir level will depend on preoperative baseline PSA. Additionally, it is well known that the baseline PSA is influenced by prostate size, urinary retention status, urinary infection, and presence of prostatitis or incidental Pca. Therefore, we seek to ask whether or not a patient's preoperative characteristics, including prostate size, PSA value, and other factors ultimately influence their post‐HoLEP PSA nadir and should we expect a standardized baseline regardless of preoperative variables affecting PSA values?

We investigated the relationship of preoperative prostate size, urinary retention status, positive urine culture, histopathological evidence of prostatitis, and incidental Pca on baseline and 3‐month follow‐up nadir PSA value after HoLEP. Since knowledge of nadir PSA at 3 months plays a critical role in prostate cancer screening, it is vital to understand factors that might influence this level. To the best of our knowledge, this study is first to evaluate the impact of patient‐related perioperative variables on post‐HoLEP nadir PSA value. We also reviewed the literature to determine the impact of common surgical techniques: transurethral resection of prostate (TURP), open prostatectomy (OP), and various endoscopic enucleation procedures on nadir PSA values.

## 2 PATIENTS AND METHODS

### 2.1 Patient selection

This study included patients who underwent En‐bloc HoLEP at our institution from July 2017 to June 2019. Patient data were prospectively collected and retrospectively analyzed (Table 1). Institutional Review Board approval was obtained. Elevated PSA was evaluated before HoLEP with imaging, 4K score, and prostate biopsy, as indicated after shared decision making. Since the aim of the present study was to look for a nadir PSA level which may help in prostate cancer screening after HoLEP, we excluded patients with preoperative diagnosis of prostate cancer. Similarly, patients with post‐HoLEP symptomatic UTI, and those with missing 3‐month PSA data were also excluded (Table 1).

**TABLE 1:** Patient characteristics and details of patients excluded from analysis

Patients characteristics	
Parameters	Number (%)
Total patients	90
Patients age (years)	69.19 ± 7.21
Median (Min, max)	70 (53‐90)
Presence of median lobe	
Yes	64
No	26
Preoperative urinary retention status	
Yes	64 (71.1%)
No	26 (28.9%)
Preoperative urine culture status	
Positive	22 (24.4%)
Negative	68 (75.6%)
Weight of resected tissue (gram)	
<40	12 (13.3%)
40‐80	16 (17.8%)
>80	62 (68.9%)
Median (Min, max)	119.8 (3.3, 375)
Mean ± SD	165.9 ± 144.7
Histopathological diagnosis	
BPH	71 (78.9%)
BPH + INF	12 (13.3%)
BPH + PCA	7 (7.8%)
Gleason Group 1	5 (2.2%)
Gleason Group 2	2 (5.6%)
Finasteride Exposure	
Yes	38 (42.2%)
No	52 (57.8%)
Prior Prostate Biopsy	
Yes	32 (35.6%)
No	58 (64.4%)
PSA at Baseline	
<4	38 (42.2)
4‐8	27 (30.0)
>8	25 (27.8)
Mean ± SD	6.3 ± 5.9
Median (Min, max)	4.7 (0.4, 39.1)
PSA at 3‐Month Post‐HOLEP (N = 90)	
Mean ± SD	0.6 ± 0.6
Median (Min, max)	0.4 (0.1, 3.6)
PSA at 6‐Month Post‐HOLEP (N = 25)	
Mean ± SD	0.82 ± 0.72
Median (Min, max)	0.70 (0.37, 0.90)
PSA at 12‐Month Post‐HOLEP (N = 42)	
Mean ± SD	0.58 ± 0.73
Median (Min, max)	0.40 (0.28, 0.76)
PSA Percentage decrease at 3 months	
Mean ± SD (%)	85.6 ± 16.7
Median (Min, max) %	91.4 (12.3, 99.7)
*Details of patients excluded from analysis*	
Preoperative diagnosis of prostate cancer	19
Patient undergoing HoLEP + HIFU for treatment of prostate cancer	5
Patient developed refractory urine retention after radiotherapy for prostate cancer	2
Patient undergoing HoLEP prior to radiotherapy	1
Castrate‐resistant prostate cancer with urine retention or gross hematuria	3
Hx of proton beam therapy for prostate cancer with bothersome LUTS	1
Hx of PCa on active surveillance	7
Patient with preoperative diagnosis of prostatic abscess	2
Patient with Epididimo‐orchitis at 2‐month post‐HoLEP	1
Patient missing 3‐month PSA data^a^	49

^a^ Most of these patients had PSA within 2 months of HoLEP or after 4 months of HoLEP and hence these patients were excluded from the present study.

### 2.2 Intervention

All procedures were performed by a single, experienced surgeon. The procedure was performed using a Holmium laser machine at settings of 2 J and 30 Hz for the entire procedure. After an initial cystoscopy, an inverted U‐shaped incision was made in the mucosa proximal to the verumontanum. This incision was extended laterally to enter the plane of enucleation. The adenoma was dissected from the pseudo‐capsule counter‐clockwise from 5 to 9 o'clock using a combination of blunt dissection and holmium laser energy. The vertical fibers near the bladder neck were incised at 12 o'clock anterior to the adenoma to enter the bladder. Thereafter, both lateral lobes were dissected from the bladder neck using laser energy. The right lobe apex was then dissected in a clockwise direction to connect the plane of enucleation that was developed from the anterior aspect of the right lateral lobe. At this point, the entire prostate adenoma typically remains attached to the membranous urethra anteriorly. This antero‐apical mucosal strip was then incised with the aim of safeguarding the sphincter. Finally, the prostate was separated from its posterior capsule, and pushed into the bladder for morcellation.

### 2.3 Outcomes

The primary outcome was to assess the effect of preoperative urinary retention status, preoperative urine culture status, amount of enucleated tissue, and histopathologic diagnosis (BPH, BPH with prostatitis, and BPH with Pca) on baseline PSA and 3‐month post‐HoLEP nadir PSA level. PSA data at 6 month and 1 year were also reviewed. Since the preoperative measurement of prostate volume was not standardized, and measurements were done by various modalities that include transrectal ultrasound, CT scan, MRI, transabdominal ultrasound, or estimation on DRE, we did not use that data of preoperative prostate weight for analysis. Instead, we choose to use data of enucleated prostate weight which was measured by a single pathologist in all patients as a surrogate marker of preoperative prostate size.

### 2.4 Statistics

Descriptive statistics were calculated to summarize the distribution of patient variables. We performed univariable and multivariable analyses to assess the effect of perioperative variables on predicting PSA at baseline, PSA at 3 months after HoLEP, and PSA decrease. The distributions of PSA outcomes did not meet the criteria of normal distribution by the Shapiro‐Wilk test. We tested fit of alternative distribution and found that the log‐normal and gamma distributions fitted the data similarly well. Therefore, we used paired Student *t* tests to compare log‐transformed PSA baseline and at 3 months. The evaluation of potential predictors of a particular PSA outcome was made through fit of generalized linear models (GLMs) under gamma distribution and with natural log link function for each particular PSA outcome. The postoperative PSA at 3, 6, and 12 months were compared by Kruskal‐Wallis test. The significance level was set as .05. Analyses were performed in SAS v9.4.

## 3 RESULTS

**FIGURE 1:**
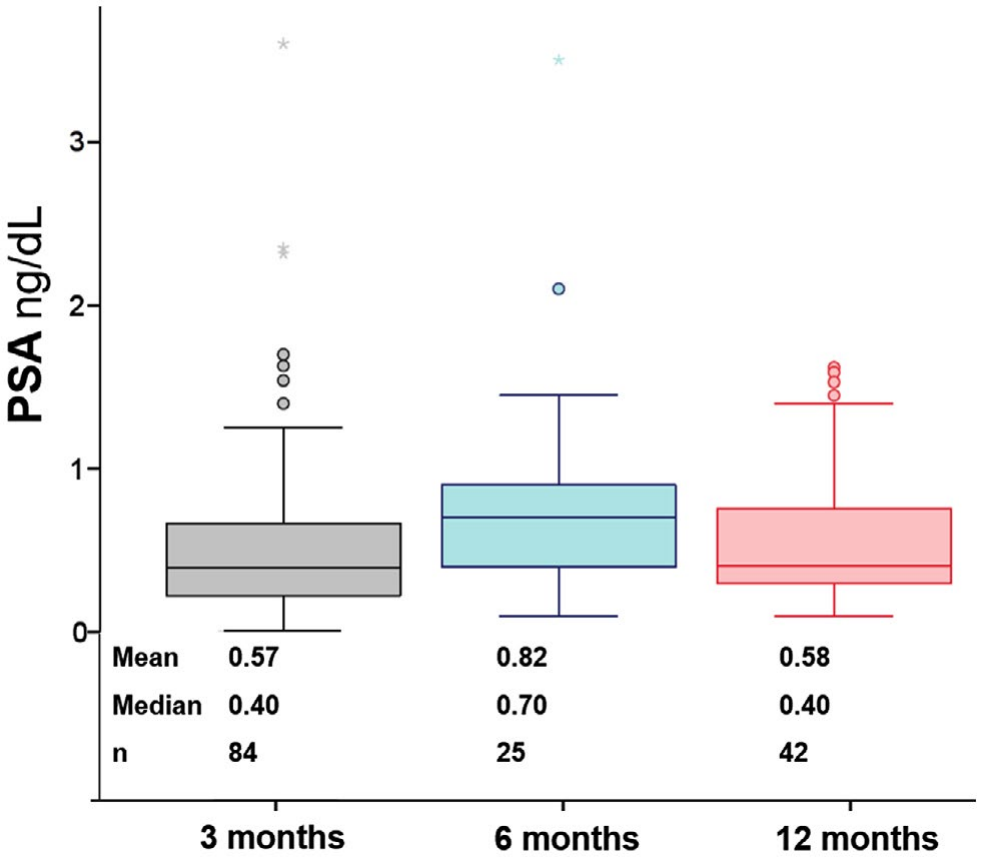
Postoperative PSA at 3, 6, and 12 months after En‐bloc HoLEP. (*P* = .060, Kruskal‐Wallis test)

During the study period, 161 patients underwent HoLEP and 90 patients met inclusion criteria for this study. Patient characteristics are displayed in supplementary Table S1. Mean PSA at baseline and 3 months postoperatively were 6.3 ± 5.9 ng/mL and 0.6 ± 0.6 ng/mL, respectively. This change was statistically significant (*P* < .0001) and corresponded to a PSA dropped on average by 85.6% (range from 12.3% to 99.7%) from baseline to 3 months post En‐bloc HoLEP. Moreover, 76 (84.4%) patients had PSA <1 at 3 months post‐HoLEP. A subset of 25 patients had PSA of 0.82 ± 0.72 ng/dL (baseline PSA = 6.29 ± 4.07 ng/dL) at 6 months follow‐up. One‐year follow‐up PSA data available from 42 patients revealed that PSA continued to remain low (0.58 + 0.73 ng/dL). (Figure 1).

**FIGURE 2:**
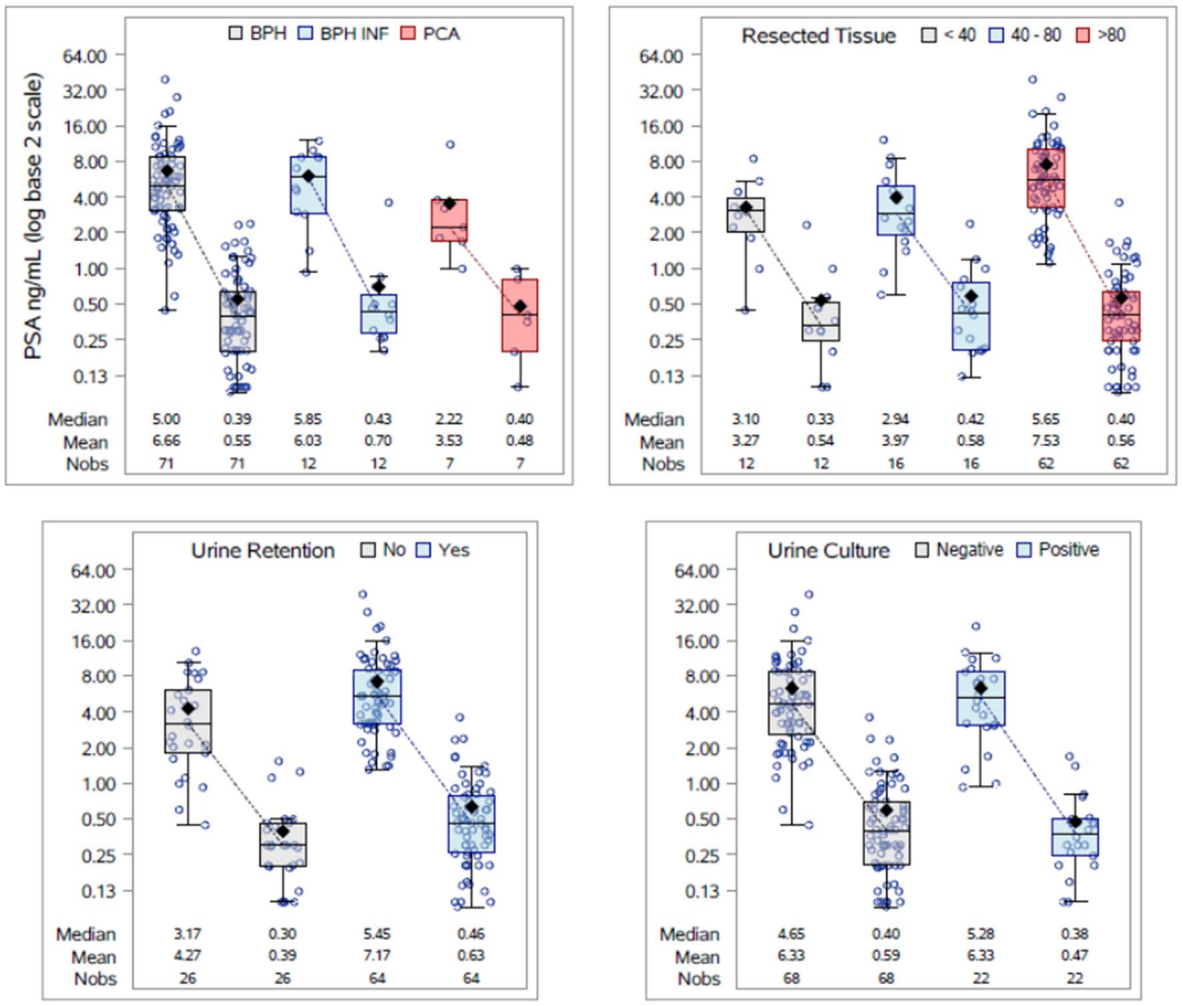
PSA (ng/mL) at baseline and 3 months after HoLEP. Significant reductions in PSA at 3 months post‐HoLEP in all subsets of patients defined by histopathological diagnosis, resected tissue weight, urinary retention status, and urine culture result (*P* < .0001, paired *t* tests of log‐transformed data)

There were significant reductions in PSA at 3 months post‐HoLEP in all subsets of patients (*P* < .001) (Figure 2). The amount of resected tissue and urinary retention status were significant predictors of baseline PSA, but only urinary retention status was a significant predictor of PSA at 3 months post‐HoLEP. With respect to PSA at baseline, patients who had a larger amount of resected tissue or patients with urinary retention had higher baseline PSA values (Table 2). For PSA at 3 months post‐HoLEP, patients with urinary retention had higher postoperative PSA values. Of note, baseline PSA (*P* = .281), histopathological group (*P* = .724), urine culture status (*P* = .158), and weight of resected tissue (*P* = 8.65) had no significant effect on PSA at 3 months post‐HoLEP (Table 2). We noted that patients with smaller prostate having resected tissue weight <40 g had a lower baseline PSA and decreased mean percentile reduction in PSA when compared with those with larger prostate having resected tissue weight >80 g (mean baseline PSA of 3.27 vs 7.53 ng/dL; *P* = .002 and percentile reduction of 77.67% vs 89.06%; *P* < .001). Additionally, patients with small vs large prostate noted similar PSA nadir level at 3 month (0.54 vs 0.56 ng/dL).

**TABLE 2:** Univariable and multivariable analysis for predictors of PSA baseline and at 3 months post‐HoLEP procedure

Variable	Category	PSA at baseline		PSA at 3 months		PSA drop		Percentage PSA drop	
		Mean (95%CI)	*P*	Mean (95%CI)	*P*	Mean (95%CI)	*P*	Mean (95%CI)	*P*
*Univariable analysis*									
Diagnosis group	BPH	6.66 (5.57, 7.95)	.157	0.55 (0.46, 0.66)	0.520	6.11 (5.00, 7.46)	.175	86.13 (80.99, 91.59)	.796
	BPH INF	6.03 (3.92, 9.29)		0.70 (0.45, 1.09)		5.33 (3.28, 8.68)		85.53 (73.64, 99.33)	
	PCA	3.53 (2.01, 6.21)		0.48 (0.27, 0.86)		3.05 (1.61, 5.78)		80.19 (65.92, 97.54)	
Resected tissue	< 40	**3.27 (1.75, 6.11)**	**.002**	0.54 (0.34, 0.84)	0.967	**2.73 (1.73, 4.33)**	**<.001**	77.67 (67.12, 89.88)	.083
	40‐80	**3.97 (2.42, 6.49)**		0.58 (0.39, 0.86)		**3.38 (2.27, 5.04)**		78.07 (68.80, 88.59)	
	>80	**7.53 (6.28, 9.03)**		0.56 (0.46, 0.69)		**6.97 (5.69, 8.53)**		89.06 (83.52, 94.96)	
Urinary Retention	No	**4.27 (3.20, 5.69)**	**.005**	**0.39 (0.29, 0.53)**	**0.012**	**3.87 (2.79, 5.37)**	**.012**	83.84 (75.72, 92.82)	.639
	Yes	**7.17 (5.97, 8.61)**		**0.63 (0.52, 0.76)**		**6.54 (5.31, 8.05)**		86.30 (80.88, 92.08)	
Urine culture	Negative	6.33 (5.26, 7.61)	.998	0.59 (0.49, 0.72)	0.257	5.74 (4.66, 7.06)	.925	84.71 (79.55, 90.20)	.521
	Positive	6.33 (4.58, 8.75)		0.47 (0.34, 0.66)		5.86 (4.06, 8.44)		88.30 (79.06, 98.62)	
*Multivariable analysis*									
Diagnosis group	BPH	4.46 (3.52, 5.66)	.248	0.47 (0.36, 0.61)	0.724	3.98 (3.03, 5.22)	.266	82.75 (75.84, 90.30)	.908
	BPH INF	4.33 (2.83, 6.62)		0.55 (0.34, 0.89)		3.81 (2.34, 6.18)		83.59 (71.55, 97.67)	
	PCA	2.67 (1.58, 4.52)		0.42 (0.23, 0.76)		2.23 (1.22, 4.08)		79.21 (65.16, 96.30)	
Resected tissue	< 40	**3.01 (1.96, 4.63)**	**.002**	0.47 (0.29, 0.78)	0.865	**2.55 (1.56, 4.17)**	**.002**	78.75 (67.40, 92.02)	.108
	40‐80	**3.05 (2.07, 4.50)**		0.51 (0.33, 0.78)		**2.57 (1.64, 4.02)**		77.92 (67.52, 89.91)	
	>80	**5.62 (4.25, 7.43)**		0.45 (0.33, 0.61)		**5.16 (3.75, 7.10)**		89.30 (80.28, 99.33)	
Urinary Retention	No	**3.01 (2.16, 4.20)**	**.015**	**0.38 (0.26, 0.57)**	**0.021**	**2.66 (1.82, 3.88)**	**.050**	82.06 (72.65, 92.69)	.928
	Yes	**4.60 (3.52, 6.01)**		**0.59 (0.45, 0.79)**		**3.94 (2.89, 5.36)**		81.60 (73.89, 90.11)	
Urine culture	Negative	3.80 (2.93, 4.93)	.823	0.55 (0.40, 0.75)	0.158	3.26 (2.42, 4.39)	.936	80.00 (72.64, 88.11)	.480
	Positive	3.65 (2.58, 5.16)		0.42 (0.28, 0.61)		3.21 (2.16, 4.77)		83.70 (73.80, 94.94)	

Note

Mean (95%CI): Unadjusted or adjusted predicted means with corresponding 95% confidence intervals from generalized linear models under gamma distribution and with natural log link function for the particular outcome variable. P: *P*‐value testing significance of the variable in the model.

The multivariable model for PSA at 3 months includes the baseline PSA as a continuous variable, even thought it was not statistically significant (*P* = .281).

Upon review of literature, we noted that nadir PSA levels reported post‐HoLEP are much lower than most series of TURP and simple prostatectomy (Table 3). In the reported case series following endoscopic prostate enucleation, PSA nadir ranged from 0.5 to 1.9 ng/mL and % PSA decline ranged from 61% to 89%. The overall mean post‐procedure PSA value was lower with the En‐bloc technique than with the traditional two‐lobe or three‐lobe techniques (Table 3).

**TABLE 3:** Review of literature showing decrease in PSA with TURP, Open prostatectomy, and various endoscopic enucleation procedures for BPH

Author/year	Number of patients	Prostate size Mean ± SD Or median (range)	Baseline PSA	Interval after surgery PSA estimation	Postoperative PSA	% drop
*Open prostatectomy*						
Stamey et al.^4^ 1987	7	N/A	24	3 weeks	1	96%
Scattoni et al.^20^ 1999	44	83.9	6.11	3 months	1.14	81.3%
Helfand et al.^12^ 2009	68	148.5 ± 64.5	12.9 ± 4.6	< 1 year	1.0 ± 0.9	92.9%
Rao et al.^21^ 2013	40	110.2 ± 32.1	4.52 ± 2.14	3 months	1.24 ± 0.71	72.6%
		75.2 ± 20.4^#^		6 months	0.58 ± 0.47	87.2%
				1 year	0.61 ± 0.49	86.5%
*TURP*						
Stamey et al.^4^ 1987	73	29 ± 19	7.9 ± 7.1	unclear	1.3 ± 1.5	84%
		(6‐104)^#^				
Oesterling et al.^22^ 1993	13	N/A	6.8 (0.5‐22.8) *	18 day (12‐30+)	0.7 (0.2‐8.2) *	90%
Aus et al.^23^ 1996	190	33.5 (4‐138)^#^	6.0 ± 7.7	3‐4 month	1.9 ± 2.5	69.7%
		63.3 (10‐195)				
Marks et al.^5^ 1996	82	N/A	4.62	6 months	0.85	81.6%
Recker et al.^24^ 1998	NC‐96	39.6 ± 18.3	4.71 ± 4.29	3‐4 month	1.75 ± 2.21	63%
	C‐19	49.3 ± 21.4	8.45 ± 5.14		2.86 ± 3.29	66%
Shingleton et al.^25^ 2000	50	29.6 ± 2.2	3.2 ± 0.31	1 year	1.7 ± 0.22	46%
Fonseca et al.^1^ 2008	30	71.8 ± 24.0	6.19 ± 7.06	1 month	2.27 ± 2.20	63%
		29.87 ± 19.58^#^		2 months	1.75 ± 1.66	71%
				6 months	1.79 ± 1.26	71%
Helfand et al.^12^ 2009	343	51.2 ± 34.7	4.2 ± 1.4	<1 year	1.6 ± 1.8	61.9%
*HoLEP‐two‐lobe or three‐lobe techniques*						
Elzayat et al.^7^ 2007	118	53.3 (20‐172 cc)	5.8 ± 4.9	6 months	1.9 + 2.1	67
Elmansy et al.^6^ 2009	326	81.976 ± 43.81	5.44 ± 5.15	3 months	0.91 ± 1.05	75.39
Tinmouth et al.^8^ 2005	McGill‐323	79.0 (13‐305)	6.0 (0.12‐41.4)	6 months	1.1 (0.05‐22.1)	81.7
		49.8(5‐300)^#^				
		111.9 (15‐309.5)				86.0
	Methodist‐186	90.4 (8‐312)^#^	8.6 (0.4‐120.0		1.2 (0.01‐12.0)	
Otsubo et al.^18^ 2015	BPH‐340	55.5 (15‐230)	4.5 (0.43‐34.08)	12.2 (4‐54)* months	0.75 (0.1‐7.16)	83.2
	Pca‐25	47 (25‐100)	7.14 (1.26‐373)	31.5 (14‐59)* months	1 (0.14‐6.44)	83.2
Gilling et al.^26^ 2008	34	27.2 + 25.2	4.6 ± 5.2	6.1 years	1.8 ± 1.3	61
Krambeck et al.^15^ 2010	83			>5 year	0.95 (0.029‐8.13)	
Ibrahim et al.^16^ 2019	132	92.3 ± 51.5 *	6.1 ± 4.4	12.6 (10‐18) year*	1.7 ± 2.0	66.7
*En‐bloc HoLEP*						
Saitta et al.^10^ 2019	137	75.63 ± 42.1	4.8 ± 7.00 (3‐70)	3 months	0.75	84
				6 months	1.25	74
				12 months	1.06	78
*ThuLEP*						
Kim et al.^11^ 2015	47	66.9 ± 36.6	7.8 ± 15.9	1 month	0.5 ± 0.4	
Castellani et al.^27^ 2018	Vanese‐82 Ancona‐82	56.78 ± 20.8	4.1 (2.7;6.9)^	6 months	0.6 (0.4;0.9)^	85
		56.89 ± 20.5	3.4 (1.7;5.8)^		1 (0.6; 1.5) ^	71
Enikeev et al. 2019^28^ Combined with HoLEP	En‐bloc‐406	86.11	4.7 ± 3.9	≤ 6 months	0.9 ± 0.3	81
	Two‐lobe‐709	87.70	4.8 ± 4.2		0.9 ± 0.3	81
Morozov et al. 2020^14^ With HoLEP and MEP	HoLEP‐509	91 ± 44	4.5 ± 3.1	6 months	1.0 ± 0.3	78
	Thulep‐812	86 ± 40	5.2 ± 5		1.0 ± 0.3	81
	MEP‐92	68 ± 23	3.8 ± 2.2		1.0 ± 0.3	74
*PKEP*						
Rao et al.^21^ 2013	43	65.9 ± 20.8^#^	4.77 + 2.21	3 months	1.18 + 0.69	75
				6 months	0.54 + 0.49	89
				12 months	0.55 + 0.43	88

Note

#, resected tissue weight; *, median (range); ^, median (IQR); ThuLEP‐Thulium laser enucleation of prostate; and MEP‐Monopolar Enucleation of Prostate.

## 4 DISCUSSION

HoLEP is recommended as a size‐independent procedure for the treatment of an enlarged prostate by the American Urology Association. We employed an En‐bloc technique for HoLEP in present study and noted an average 3‐month postoperative PSA valve of 0.6 ± 0.6 ng/mL, with 85.6 ± 16.7% decrease from baseline. The only other study examining PSA nadir after En‐bloc HoLEP reported a similar PSA decrease of 84% with an average 3‐month postoperative PSA nadir of 0.75 ng/dL.^10^ Kim et al. described a similar “all‐in‐one” En‐bloc technique of enucleation using the Thulium laser and found an 81% reduction in PSA from a baseline level from 7.8 ± 15.9 to 0.5 ± 0.4 at 1 month after surgery.^11^ However, three other studies that measured nadir PSA at 3‐6 months post‐HoLEP with the traditional two‐lobe or three‐lobe techniques found a nadir level ranging from 0.9 to 1.9 ng/dL.^6^, ^7^, ^8^ Employing an En‐bloc technique, we and other authors noted significantly lower nadir PSA level at 3 months when compared with those reported in literature with the traditional two‐lobe or three‐lobe techniques (Table 3). The influence of evolving techniques and increasing experience on nadir PSA level after procedure is evident from a series of publications from McGill University. When these authors analyzed the results of HoLEP in their first 118 patients they noted that the PSA level dropped at 6 months by 67.3% from 5.8 ± 4.9 to 1.9 ± 2.1.^7^ After a decade of performing HoLEP, the nadir PSA level dropped to 0.91 ± 1.05.^6^

Traditionally, it is believed that each gram of tissue removed during TURP causes a reduction in PSA by 0.1‐0.3 ng/mL.^12^ However, our study indicates that this is no longer applicable following complete enucleation. On sub‐group analysis, we also noted that although patients with smaller prostates (resected tissue weight <40 g) had a smaller percentile reduction in PSA when compared with those with larger prostates (resected tissue weight >80 g) (77.67% vs 89.06%; *P* < .001), patients from both these groups noted a similar PSA nadir level at 3 month (0.54 vs 0.56 ng/dL) (Figure 2). After complete adenomectomy, the residual peripheral zone remains the only source of PSA. It has been shown that both the transition zone and peripheral zone of the prostate grows with age, but once the total prostate volume exceeds 30 g, the size of the peripheral zone becomes attenuated.^13^ As most of the patients in our study had prostate sizes >30 gm, we believe that the volume of peripheral zones were equivalent in these patients, resulting in similar PSA values after HoLEP.

PSA nadir is also independent of use of holmium laser, thulium laser, or monopolar energy sources for endoscopic enucleation^14^ after complete adenomectomy, dramatic reduction in PSA velocity is also expected. We noted that PSA level remained stable up to 1‐year follow‐up. Other authors also noted mean PSA level of 0.95 at follow‐up of >5 years after HoLEP.^15^ At a median follow‐up of 12.6 years, the PSA decrease continued to remain at 66.7% from its pre‐HoLEP level.^16^ PSA velocity is high in patients diagnosed with Pca during follow‐up after HoLEP and a threshold of 0.38 ng/mL/y was found to be highly specific for detecting Pca.^6^, ^12^

In our study, patients with preoperative urinary retention were noted to have higher postoperative PSA values. The impact of preoperative urinary retention on postoperative nadir PSA at 3 months was surprising as previously published literature demonstrated that PSA returns to baseline after 2 weeks of drainage via catheterization.^17^ Although the difference was statistically significant, we believe that a difference in post‐HoLEP PSA of 0.57 vs 0.37 may not be clinically significant. Additionally, preoperative urine culture status was not found to affect either baseline PSA level or postoperative PSA nadir at 3 months.

We did not detect statistically significant differences in nadir PSA level at 3 months between patients with a histopathological diagnosis of Pca, prostatitis, or benign prostatic hyperplasia. Baseline PSA level in patient with histological evidence of prostatitis was found similar to those without prostatitis (6.03 vs 6.66 *P* = .157) and both groups had similar mean nadir PSA level at 3‐month after surgery (0.70 vs 0.55; *P* = .520). Similarly, in one study following TURP, there was no significant difference between patients diagnosed with and without prostatitis on postoperative PSA.^1^ It is surprising that presence of PCa in a specimen after HoLEP did not lead to higher post‐HoLEP PSA values. Of note, this group was also not found to have a higher baseline PSA, suggesting that these individuals may have clinically indolent PCa. This is consistent with our findings that these patients had low to favorable intermediate risk PCa. A total of 35.6% of the patients in our study had at least one negative prostate biopsy before HoLEP. Similar to our findings, Otsubo et al. reported that patients diagnosed with incidental Pca had a similar reduction (83.2%) in PSA after HoLEP.^18^

Based on our findings, we believe that if Pca is not detected on histopathology evaluation after HoLEP and the post‐HoLEP nadir PSA is significantly higher, that patient should be counseled about further evaluation targeted toward early detection of Pca, especially if a complete adenomectomy was performed. In a recent publication, patients diagnosed with Pca during follow‐up period post‐HoLEP had a higher median PSA at first post‐HoLEP follow‐up compared with those who did not undergo prostate biopsy (1.6 vs 0.68).^9^ Authors noted that patients with a post‐HoLEP PSA above 1 ng/mL had a 94% probability of cancer detection and an 80% risk of clinically significant disease and hence recommended prostatic biopsy for all men with post‐HoLEP PSA above 1 ng/dL.^9^ Similarly, since TURP involves less removal of adenoma compared to HoLEP, Wolff et al. noted that patients who develop Pca after TURP for BPH had a postoperative nadir PSA above 2 ng/mL.^19^ They suggest that patients with nadir PSA >2, or those having an early rise in PSA after TURP, should be evaluated with a high index of suspicion for Pca.

The primary limitation of this study is its retrospective, non‐comparative nature and that interventions were all performed by a single experienced surgeon in a tertiary referral center thereby limiting the generalizability of our findings. We also did not evaluate correlation between the drop in PSA with the outcome of surgery. Shorter follow‐up and fewer number of patients prohibit us from making any meaningful conclusion of long‐term cancer behavior in patients diagnosed incidentally with prostate cancer. Despite this, our study indicates that post‐HoLEP PSA nadirs are independent of any patients‐related factors. Our findings can be generalized to state that nadir PSA level after any surgical procedure for BPH should be independent of any patient‐related factor and should depend only on non‐patient‐related factors like technique and completeness of removal of transition zone as confirmed on our review of literature.

We conclude that patients are expected to have a similar PSA nadir at 3 month after HoLEP, regardless of preoperative factors. We found that PSA levels following HoLEP are independent of evidence of indolent Pca or prostatitis on histopathologic examination and remains stable up to 12‐months follow‐up period. We recommend that Pca surveillance patients should have a PSA measurement at 3 months after any surgical intervention for BPH to evaluate the new PSA nadir.

## ETHICS APPROVAL

The study was approved by University of Miami Ethics Committee‐Study ID‐20,180,511.

## CONSENT TO PARTICIPATE

Every patient consented to participate in study.

## CONSENT FOR PUBLICATION

Every patient consented to participate in study.

## CONFLICT OF INTEREST

The authors declare no competing financial interests.

## AUTHOR CONTRIBUTIONS

Martos, Mary Patricia—writing original draft and investigation. Jonathan E. Katz—supervision, investigation, and project administration. Parmar, Madhumita—investigation. Jain, Anika—investigation. Nachiketh Soodana‐Prakash—resources and data curation. Punnen, Sanoj—writing review and editing, resources. Gonzalgo, Mark L.—writing review and editing, resources. Reis, Isildinha M.—formal analysis, writing review and editing. Smith Nicholas A.—writing review and editing. Shah Hemendra Navinchandra‐conceptualization, methology, visualization, resources, writing review and editing.

## Data Availability

The raw data can be made available at request after getting approval from University of Miami Ethics Committee.
